# Health literacy of recently hospitalised patients: a cross-sectional survey using the Health Literacy Questionnaire (HLQ)

**DOI:** 10.1186/s12913-016-1973-6

**Published:** 2017-01-19

**Authors:** Rebecca L. Jessup, Richard H. Osborne, Alison Beauchamp, Allison Bourne, Rachelle Buchbinder

**Affiliations:** 1 Deakin University, Health Systems Improvement Unit, Centre for Population Health Research, School of Health and Social Development, Geelong, Victoria 3220 Australia; 2 Monash Department of Clinical Epidemiology, Cabrini Institute, Malvern, VIC 3144 Australia; 3 Department of Epidemiology and Preventive Medicine, School of Public Health and Preventive Medicine, Monash University, Suite 41 Cabrini Medical Centre, 183 Wattletree Road, Malvern, VIC 3144 Australia

**Keywords:** Health literacy, Hospitalisation, Equity, Access

## Abstract

**Background:**

Health literacy is simply defined as an individual’s ability to access, understand and use information in ways that promote and maintain good health. Lower health literacy has been found to be associated with increased emergency department presentations and potentially avoidable hospitalisations. This study aimed to determine the health literacy of hospital inpatients, and to examine if associations exist between different dimensions of their health literacy, sociodemographic characteristics and hospital services use.

**Methods:**

A written survey was sent to 3,252 people aged ≥18 years in English, Arabic, Chinese, Vietnamese, Italian or Greek. The survey included demographic and health questions, and the Health Literacy Questionnaire (HLQ). The HLQ is a multidimensional instrument comprising nine independent scales. Use of hospital services was measured by length of stay, number of admissions in 12 months and number of emergency department presentations. Effect size (ES) for standardised differences in means described the magnitude of differences in HLQ scale scores between demographic and socioeconomic groups.

**Results:**

385 questionnaires were returned (13%); mean age 64 years (SD 17), 49% female. Aged ≥65 years (55%), using the Internet < once a month (37%), failure to complete high school (67%), low household income (39%), receiving means-tested government benefits (61%) and being from a culturally and linguistically diverse (CALD) background (24%), were all associated with lower scores in some health literacy scales. Being aged ≥65 years, not currently employed, receiving government benefits, and being from a CALD background were also associated with increased use of some hospital services. There was no association between lower scores on any HLQ scale and greater use of hospital services.

**Conclusion:**

We found no association between lower health literacy and greater use of hospital health services. However increased age, having a CALD background and not speaking English at home were all associated with having the most health literacy challenges Strategies to address these are needed to reduce health inequalities.

## Background

The provision of safe, equitable and accessible health care is an ongoing challenge for all health service providers. In the face of increasing pressure from an ageing population [1, 2], growth in chronic and preventable disease [3], increasing healthcare costs and workforce shortages [4–8], and changing community expectations [9, 10] hospitals are under unprecedented pressure to deliver the right care, at the right time. An ongoing challenge for hospitals is the management of potentially avoidable hospitalisations for conditions that may have been treated or managed out of hospital [11]. While the reasons for requiring hospitalisation are complex and multifactorial, addressing a patient’s health literacy needs may be one potential strategy for reducing avoidable hospitalisations.

Health literacy is defined by the World Health Organisation as ‘the cognitive and social skills which determine the motivation and ability of individuals to gain access to, understand and use information in ways which promote and maintain good health’ [12]. Individuals require good health literacy in order to access and understand all the information and support they require to appropriately manage unexpected acute illness or existing chronic conditions [13, 14].

Several studies have found an association between lower health literacy and potentially avoidable hospitalisations [15–17]. However these studies have generally used health literacy tools that are unidimensional, and only capture one aspect of health literacy, i.e. health-related reading +/− numeracy ability [18]. As implied by the definition above and empirical data [19], health literacy is a multidimensional concept that cannot be fully captured by a single skill or attribute. The influence of contextual, social and cultural factors, and the ability of healthcare practitioners and healthcare organisations to meet patients’ health literacy needs have received little attention.

The Health Literacy Questionnaire (HLQ) is a multidimensional instrument designed to generate a profile of an individual’s or population’s health literacy strengths and weaknesses [20]. It comprises 44 items across nine independent scales: *Feeling understood and supported by healthcare providers; Having sufficient information to manage my health; Actively managing my health; Social support for health; Appraisal of health information; Ability to actively engage with healthcare providers; Navigating the healthcare system; Ability to find good health information; and Understanding health information enough to know what to do*. In contrast to single dimensional measures, the HLQ has been demonstrated to provide a detailed profile of an individual’s health literacy skills and needs. It has also been used to provide guidance for the development of interventions to address these needs [21], and therefore may also be useful to guide development of interventions to reduce avoidable hospitalisations. The aim of this study was to determine the health literacy strengths and weaknesses of a cohort of hospital inpatients, and to examine if associations exist between the different dimensions of health literacy and patients’ demographic and socioeconomic characteristics and use of hospital services.

## Methods

A cross-sectional survey was posted to patients who had recently (less than 30 days) attended an acute public hospital in Victoria, Australia. This hospital is located in Melbourne’s northern suburbs and provides care to a diverse community. The hospital’s catchment includes a higher proportion of people and households with lower income, lower educational attainment, higher numbers of migrants, and higher rates of unemployment than Victorian State averages [22]. The utilisation rates for hospital interpreter services identifies that over 120 languages are spoken amongst patients attending this service.

Data collection took place over six months from January to June 2015. Each month, 500 participants were invited to participate. Potential participants were identified through the hospital’s computerized clinical and administrative data warehouse. Participants were eligible if they had been hospitalised for at least 24 h in the past 30 days, and if they spoke English, Arabic, Chinese, Vietnamese, Greek or Italian, all languages in the top 10 most spoken languages in the region. Participants were excluded if they had been hospitalised for less than 24 h to ensure that we excluded patients admitted for day medical procedures, day oncology, dialysis or those briefly attending emergency. Participants were also excluded if they were aged under 18, had a history of cognitive impairment, or if they were discharged to another institution rather than returning home. Each month, purposive sampling was used to identify all patients who spoke one of the non-English target languages to ensure oversampling of these groups. The remaining participants were randomly selected from eligible English speaking patients.

To maximise the survey response rate, each participant was sent a pre-notification letter written in their language and signed by the hospital’s Chief Executive Officer (CEO). The letter encouraged participation in order to assist the hospital to improve services. A week later, participants were mailed the participant information and consent form, the HLQ and a brief survey of health and demographic and socio-economic variables in their language. A late return/reminder letter was sent two weeks following the mail out of the survey.

The study was approved by the Northern Health and Deakin University Human Research and Ethics Committees.

### Hypotheses

In keeping with previous studies, we hypothesised that being older, not speaking English at home, being born in a country where English was not the first language, and/or living alone would be associated with lower scores across all nine HLQ scales [21]. We also hypothesised an association between lower scores across the nine HLQ scales and lower socioeconomic status (unemployment, leaving school before completion, household income less than $30,000, on government means tested benefits, not having private health insurance) and minimal Internet use (less than once a month).

With respect to use of hospital services, we hypothesized an association between lower health literacy and indicators of lower socioeconomic status and higher self-reported attendance at any hospital ED for non-urgent conditions in the previous 12 months, increased ED presentations for non-urgent conditions at the same hospital in the last 12 months, increased number of hospital admissions at the same hospital in the last 12 months, and longer length of stay for the index hospitalisation.

### Measures

Health Literacy Questionnaire: The questionnaire takes between 7 and 40 min to complete [Jessup R, Beauchamp A, Buchbinder R, Osborne R: Psychometric properties and comparability of four health literacy assessment instruments in the hospital setting, submitted]. Each domain of the HLQ consists of either 4 or 5 items. Items in the first five domains are scored from 1 to 4 (strongly disagree = 1 to strongly agree = 4), while the last four domains are scored 1 to 5 (cannot do or always difficult = 1 to very easy = 5), A domain score is calculated by adding up the scores within each domain and then dividing this value by the number of items in the respective domain with higher scores indicating higher health literacy. Each HLQ domain has been demonstrated to be conceptually distinct and measure independent constructs using confirmatory factor analysis, consequently a total score is not generated [20].

The HLQ was translated from English into Arabic, Chinese (simplified, so it was suitable for both Cantonese and Mandarin speakers), Vietnamese, Italian and Greek as these 5 languages are in the top 10 most spoken languages in the region. The translation process consisted of forward, backward then forward translation, followed by further verification with native speakers to ensure that the intended meaning of items was consistent with the item intent.

Demographic and socioeconomic data: We collected data about age, sex, living arrangements (alone or with others), indigenous status, country of birth, primary language spoken at home (English or another language), educational attainment (completion of high school or not), work status (employed or not working (unemployed, retired, ill)), household income (< or ≥ $30,000 per annum) and receipt of means-tested government benefits.

Use of hospital services: To examine use of services at the hospital where the research was based, we extracted service use information from the hospital’s data warehouse for both respondents and non-respondents. Data extracted included: whether the admission was planned (or the patient was admitted via ED), the number of days they were hospitalised, number of hospital admissions at this hospital in the last 12 months (including the index one), and number non-urgent ED presentations to this ED in the last 12 months. In addition, we also asked respondents to self-report whether they had attended any ED at any hospital in the last 12 months.

### Statistical Analysis

To determine whether an association between demographic and socioeconomic variables and hospital service use rates existed, we conducted Pearson chi-square tests using SPSS® version 22 [23]. Effect sizes were calculated using the phi coefficient, a measure of the association between two binary variables, with a small effect size classified as being between 0.10 and 0.30, a medium effect size from 0.30 to 0.50, and large effect size >0.50 [24].

Cohen’s effect sizes (ES) for standardised differences in means of HLQ scales across comparator variables were calculated using Stata® software [25]. The pooled standard deviation (PSD) was used as the denominator and difference within scales as the numerator. In this case, the effect size describes the magnitude of differences in HLQ scale scores between groups, with scores between 0.20-0.50 considered small, 0.50-0.80 considered medium, and > 0.80 considered large as described by Cohen [24]. A *p* < 0.05 was regarded as statistically significant for all tests.

We assessed the relationship between use of hospital services and demographic and socio-economic characteristics and the key outcome variable, health literacy, with the aim of advancing to multiple regression analyses if an association was found between health literacy and use of hospital services.

## Results

**Fig. 1:**
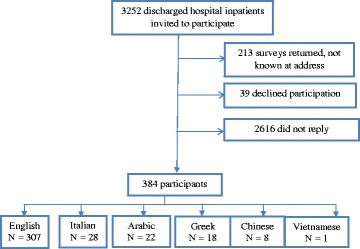
Participant flow diagram

3252 surveys were sent and responses were received from 384 participants (response rate 13%) (Fig. 1). The highest response was from participants completed the questionnaire in Chinese (n = 8/44, 18.0%), followed by English (n = 307/1981, 15.5%), Greek (n = 18/171, 10.5%), Italian (n = 28/350, 8.0%), Arabic (n = 22/314, 7.0%), and Vietnamese (n = 1/50, 2.0%).

Table 1 displays the demographic and socioeconomic details, hospital services use and reason for admission of respondents, and non-respondents where available. The mean age of participants was 64 years (SD 17) and 49% were female. By comparison, mean age of non-respondents was lower (55 years (SD 21)) but the proportion of females (51%) was similar. Study participants were ethnically diverse and there were no indigenous participants, and they generally had low income and educational attainment. Seventy (23%) lived alone, 144 (39%) were born in a country where English was not the first language, 93 (25%) did not speak English at home, and 258 (67%) had not completed high school (including 80 (21%) who completed a Trade qualification). Only 100 (26%) were currently working in any capacity (full time, part time or casually). There did not appear to be any differences between respondents and non-respondents with respect to planned admissions (50/384 (13%) versus 313/2616 (12%)), median length of hospital stay (2 days (range 1 to 87) versus 2 days (range 1 to 54)), or reasons for admission.

**Table 1:** Demographic and socioeconomic characteristics of respondents and non-respondents and their health service use

	Respondents (*N* = 384)	Non-respondents (*N* = 2616)
	*N* (%)	*N* (%)
Demographic and socioeconomic characteristics		
Female	188 (49)	1334 (51)
Age ≥ 65 years	211 (55)	1040 (40)
Lives alone (6)^a^	70 (18)	-
Did not complete high school (15)^a^	246 (67)	-
Born in English speaking country (17)^a^	144 (39)	-
English spoken at home (1)^a^	290 (76)	-
Currently employed (10)^a^	98 (26)	
Receiving government benefits (17)^a^	245 (67)	-
Private health insurance (23)^a^	111 (31)	-
Hospital service use		
Admission planned	50 (13)	313 (12)
Length of stay ≥ 7 days (2)^a^	61 (16)	348 (13)
≥ 2 admissions in last 12 months at this hospital (2)^a^	343 (90)	2276 (87)
Non-urgent ED presentations in last 12 months at this hospital (4)^a^	103 (27)	680 (26)
Reason for admission		
Maternity	7 (2)	189 (7)
Gastrointestinal	72 (19)	657 (25)
Cardiovascular	72 (19)	320 (12)
Pulmonary	45 (12)	199 (8)
Urinary	18 (5)	237 (9)
Musculoskeletal	46 (12)	302 (12)
Gynaecologic	9 (2)	61 (3)
Cellulitis	10 (3)	79 (3)
Cancer	10 (3)	82 (3)
Diabetes	15 (4)	22 (1)
Delirium	15 (4)	44 (2)

^a^Missing data

Table 2 provides an overview of the mean HLQ scale scores for participants. For the overall sample, respondents reported highest scores for *Feeling understood and supported by healthcare providers* (mean 3.13, SD 0.57) and lowest scores for *Appraisal of health information* (mean 2.82, SD 0.52). There were no appreciable differences in health literacy scores or hospital service use according to completion of the survey in English or other languages.

**Table 2:** Health literacy and health service use of participants, all and by survey language (N = 384)

	All *N* = 384	English *N* = 307	Arabic *N* = 22	Chinese *N* = 8	Greek *N* = 18	Italian *N* = 28	Vietnamese *N* = 1
	Mean (SD)	Mean (SD)	Mean (SD)	Mean (SD)	Mean (SD)	Mean (SD)	Mean
Health Literacy Questionnaire (HLQ)							
Feeling understood and supported by healthcare providers^a^	3.13 (0.57)	3.17 (0.57)	2.92 (0.50)	3.06 (0.40)	3.01 (0.33)	2.91 (0.71)	3.25
Having sufficient information to manage health^a^	2.97 (0.52)	2.98 (0.52)	2.81 (0.52)	2.91 (0.30)	2.9 (0.27)	2.98 (0.70)	3.00
Actively managing my health^a^	2.93 (0.52)	2.93 (0.52)	2.87 (0.53)	2.95 (0.54)	2.93 (0.34)	3.05 (0.61)	2.80
Social support for health^a^	3.11 (0.55)	3.12 (0.55)	3.08 (0.46)	2.93 (0.59)	3.19 (0.43)	3.06 (0.66)	2.80
Appraisal of health information^a^	2.82 (0.52)	2.81 (0.52)	2.93 (0.47)	2.9 (0.44)	2.89 (0.42)	2.83 (0.67)	3.00
Ability to actively engage with health care providers^b^	3.82 (0.78)	3.93 (0.71)	3.7 (0.64)	3.43 (0.82)	3.33 (0.95)	3.21 (1.06)	4.00
Navigating the healthcare system^b^	3.63 (0.75)	3.73 (0.69)	3.46 (0.73)	3.17 (0.79)	2.97 (0.81)	3.26 (1.02)	4.00
Ability to find good health information^b^	3.56 (0.80)	3.67 (0.73)	3.44 (0.77)	3.33 (0.87)	2.65 (0.99)	3.14 (0.95)	3.20
Reading and understanding health information enough to know what to do^b^	3.85 (0.78)	3.97 (0.67)	3.85 (0.72)	3.28 (1.00)	3.21 (0.89)	3.19 (1.12)	3.00
Hospital service use							
Length of stay*,* days	4 (8)	5 (9)	4 (5)	3 (2)	5 (4)	5 (5)	1
Number of hospital admissions in the last 12 months at this hospital	1 (1)	1 (1)	1 (0)	1 (0)	1 (1)	2 (1)	1
Number of non-urgent ED presentations in the last 12 months at this hospital	1 (4)	1 (4)	0 (1)	0 (1)	1 (1)	1 (3)	0

^a^Scale range 0–4, higher score indicates greater ability or more support

^b^Scale range 0–5, higher score indicates greater ability or more support

Table 3 explores the relationship between sociodemographic variables and use of hospital services. Aged ≥65 years was associated with being more likely to have had a hospital stay of greater than 7 days and to have two or more admissions in the last 12 months in the index hospital. Current unemployment was associated with being more likely to have two or more hospital admissions in the last 12 months in the index hospital. Lower economic status was associated with greater likelihood of two or more admissions in the last 12 months in the index hospital, having a hospital stay greater than 7 days and non-urgent ED presentations at the index hospital in the last 12 months. Not speaking English at home or coming from a non-English background was associated with a lower likelihood of self-reported non-urgent ED presentation to any hospital in the last 12 months. Having private health insurance was associated with being slightly less likely to have self-reported non-urgent ED presentation to any hospital in the last 12 months, but being more likely to have had two or more hospital admissions in the previous 12 months in the index hospital.

**Table 3:** Association between demographic and socioeconomic variables and hospital service use

	Self-reported presentation to ED in last 12 months (any hospital)	Length of stay ≥7 days	Two or more hospital admissions in last 12 months at this hospital	Two or more non-urgent ED presentations in last 12 months at this hospital
	%	%	%	%
Age				
< 65 years (*n* = 173)	90	8^a^	**18** ^**a**^	10
≥ 65 years (*N* = 211)	92	16^a^	**33** ^**a**^	13
Home living arrangement				
Lives with others (*N* = 307)	90	12	26	12
Lives alone (*N* = 70)	97	16	24	13
Employment status				
Currently employed (*N* = 113)	91	7	**17** ^**a**^	11
Currently not working (unemployed/retired/ill) (*N* = 269)	91	15	**30** ^**a**^	12
Education				
Completed secondary education or higher (*N* = 133)	93	13	28	11
Did not complete secondary education (*N* = 234)	91	13	24	17
Economic status				
Household income < $30,000 (*N* =149)	93	15	**31** ^**a**^	**17** ^**a**^
Household income ≥ $30,000 (*N* =135)	93	12	**21** ^**a**^	**4** ^**a**^
Receipt of means tested government benefits				
No (*N* = 133)	93	**16** ^**a**^	**31** ^**a**^	13
Yes (*N* = 234)	90	**8** ^**a**^	**17** ^**a**^	8
Language in country of birth				
English (Australia, New Zealand, Britain) (*N* = 219)	**96** ^**a**^	11	26	12
Non-English (*N* = 138)	**81** ^**a**^	15	27	13
Language spoken at home				
English (*N* = 283)	**96** ^**a**^	12	26	11
Another language (*N* = 90)	**78** ^**a**^	14	26	13
Use of internet				
At least once a month (*N* = 218)	93	10	25	12
Less than once a month or not at all (*N* = 139)	90	15	31	13
Private health insurance				
No (*N* = 249)	**96** ^**a**^	9	**16** ^**a**^	8
Yes (*N* = 110)	**89** ^**a**^	14	**30** ^**a**^	13

Variables in bold are significantly different between demographic or socioeconomic groups for hospital use (chi square tests *p* < 0.05)

^a^‘Small’ effect size (ES) > 0.10-0.0.30 calculated using phi co-efficient

Table 4 provides details of the associations between demographic and socio-economic characteristics and the nine scales of the HLQ. The largest differences in dimensions of health literacy were observed between participants speaking English or a language other than English at home. Compared to those who spoke English at home, speaking a language other than English at home was associated with reporting much greater difficulty *Understanding health information enough to know what to do* (ES 0.93 versus 0.65), poorer *Ability to find good health information* (ES 0.87 vs 0.73) and greater difficulty *Navigating the health care system* (ES 0.82 versus 0.70). Similar differences in mean scores on these scales were also observed between participants born in a country where English was or was not the first language. The scale that appeared most sensitive to detecting differences across sociodemographic characteristics was *Ability to find good health information*. We also found that those who rarely or never used the Internet reported finding it more difficult to *Understanding health information enough to know what to do* (ES 0.65) and poorer *Ability to find good health information* (ES 0.65). They also found it more difficult *Navigating the health care system* (ES 0.22) and *Actively engaging with healthcare providers* (ES 0.26).

**Table 4:** Association between mean HLQ scale scores and demographic and socioeconomic characteristics by effect size

		Healthcare provider support^a^	Having sufficient information^a^	Actively managing health^a^	Social support for health^a^	Active appraisal of health information^a^	Active engagement with healthcare^b^	Navigating the healthcare system^b^	Ability to find good health information^b^	Understanding health information^b^
		Mean (ES)^c^	Mean (ES)^c^	Mean (ES)^c^	Mean (ES)^c^	Mean (ES)^c^	Mean (ES)^c^	Mean (ES)^c^	Mean (ES)^c^	Mean (ES)^c^
Demographic and socioeconomic characteristics										
Sex	Female (*N* =187)	3.13	2.94	2.88	3.06	2.84	3.82	3.62	3.59	3.88
		(0.58)	(0.51)	(0.53)	(0.55)	(0.53)	(0.74)	(0.75)	(0.77)	(0.76)
	Male (*N* =194)	3.12	2.99	2.98	3.16	2.80	3.82	3.65	3.53	3.82
		(0.55)	(0.52)	(0.51)	(0.54)	(0.51)	(0.82)	(0.75)	(0.82)	(0.80)
Age, years	<65 (*N*= 166)	3.06^d^	2.91^d^	**2.87** ^d^	**3.01** ^d^	2.84	3.77	3.59	**3.66** ^d^	3.93^d^
		(0.62)	(0.55)	**(0.53)**	**(0.62)**	(0.48)	(0.79)	(0.75)	**(0.73)**	(0.69)
	≥65 (*N*= 216)	3.18^d^	3.01^d^	**2.98** ^d^	**3.18** ^d^	2.80	3.86	3.67	**3.48** ^d^	3.78^d^
		(0.51)	(0.48)	**(0.51)**	**(0.46)**	(0.55)	(0.77)	(0.75)	**(0.84)**	(0.84)
Lives alone	Yes (*N*= 70)	3.17	2.97	2.99	2.99^d^	2.77	3.76	3.60	3.39^d^	3.71^d^
		(0.54)	(0.57)	(0.59)	(0.60)	(0.57)	(0.81)	(0.85)	(0.91)	(0.84)
	No (*N*= 306)	3.11	2.96	2.91	3.13^d^	2.82	3.83	3.63	3.59^d^	3.87^d^
		(0.57)	(0.50)	(0.50)	(0.53)	(0.50)	(0.77)	(0.73)	(0.77)	(0.76)
Employed	Yes (*N*= 113)	3.09	2.98	2.93	3.13	**2.90** ^d^	3.89	3.70	**3.76** ^d^	4.00^d^
		(0.62)	(0.48)	(0.54)	(0.57)	**(0.49)**	(0.71)	(0.64)	**(0.67)**	(0.59)
	No (*N*= 268)	3.15	2.96	2.94	3.09	**2.79** ^d^	3.80	3.61	**3.47** ^d^	3.79^d^
		(0.53)	(0.53)	(0.51)	(0.54)	**(0.53)**	(0.81)	(0.79)	**(0.84)**	(0.84)
Completed secondary education or higher	Yes (*N*= 123)	3.22^d^	3.02	2.97	3.16	**2.89** ^d^	**3.97** ^d^	3.70	**3.74** ^d^	**4.08** ^d^
		(0.52)	(0.51)	(0.55)	(0.52)	**(0.50)**	**(0.67)**	(0.68)	**(0.68)**	**(0.62)**
	No (*N*= 245)	3.08^d^	2.93	2.90	3.07	**2.77** ^d^	**3.73** ^d^	3.58	**3.45** ^d^	**3.72** ^d^
		(0.58)	(0.52)	(0.51)	(0.56)	**(0.51)**	**(0.83)**	(0.78)	**(0.83)**	**(0.83)**
Household income < $30,000	Yes (*N*= 149)	3.10	2.92	2.94	3.06	2.82	**3.73** ^d^	3.54^d^	**3.40** ^d^	**3.71** ^d^
		(0.62)	(0.58)	(0.54)	(0.62)	(0.56)	**(0.90)**	(0.86)	**(0.95)**	**(0.89)**
	No (*N*= 135)	3.16	2.97	2.88	3.14	2.79	**3.92** ^d^	3.71^d^	**3.66** ^d^	**4.02** ^d^
		(0.49)	(0.48)	(0.51)	(0.48)	(0.47)	**(0.62)**	(0.61)	**(0.65)**	**(0.60)**
Receiving means tested government benefits	Yes (*N*= 233)	3.16	2.96	2.97	3.13	2.78^d^	3.81	3.62	**3.46** ^d^	**3.77** ^d^
		(0.49)	(0.51)	(0.49)	(0.50)	(0.53)	(0.77)	(0.77)	**(0.84)**	**(0.82)**
	No (*N* =133)	3.08	2.97	2.88	3.08	2.88^d^	3.84	3.64	**3.72** ^d^	**3.97** ^d^
		(0.63)	(0.52)	(0.53)	(0.59)	(0.47)	(0.77)	(0.72)	**(0.68)**	**(0.66)**
English speaking country of birth	No (*N*= 223)	**3.19** ^d^	2.99	2.94	3.13	2.83	3.95^d^	3.76^d^	**3.75** ^e^	**4.05** ^e^
		**(0.59)**	(0.52)	(0.53)	(0.57)	(0.52)	(0.72)	(0.69)	**(0.70)**	**(0.62)**
	Yes (*N*= 143)	**3.02** ^d^	2.93	2.91	3.08	2.82	3.62^d^	3.44^d^	**3.30** ^e^	**3.54** ^e^
		**(0.53**	(0.51)	(0.53)	(0.52)	(0.54)	(0.83)	(0.81)	**(0.87)**	**(0.90)**
English spoken at home	No (*N*= 92)	**3.02** ^d^	2.98	2.99	3.08	2.85	3.62^d^	3.33^e^	**3.18** ^e^	**3.35** ^f^
		**(0.47)**	(0.47)	(0.48)	(0.51)	(0.50)	(0.83)	(0.82)	**(0.87)**	**(0.93)**
	Yes (*N*= 290)	**3.15** ^d^	2.96	2.91	3.11	2.81	3.95^d^	3.73^e^	**3.68** ^e^	**4.01** ^f^
		**(0.59)**	(0.53)	(0.53)	(0.56)	(0.53)	(0.72)	(0.70)	**(0.73)**	**(0.65)**
Internet use	Yes (*N*= 219)	3.15	2.96	2.92	3.10	2.84	3.89^d^	3.69^d^	**3.76** ^e^	**4.03** ^e^
		(0.57)	(0.48)	(0.53)	(0.56)	(0.47)	(0.72)	(0.70)	**(0.64)**	**(0.61)**
	Never/rarely (*N*= 142)	3.08	2.96	2.92	3.11	2.76	3.69^d^	3.53^d^	**3.26** ^e^	**3.55** ^e^
		(0.56)	(0.58)	(0.53)	(0.54)	(0.59)	(0.87)	(0.83)	**(0.94)**	**(0.93)**
Private health insurance	Yes (*N*= 110)	3.14	3.03	2.95	3.15	2.87	3.88	3.69	3.63	3.90
		(0.60)	(0.53)	(0.55)	(0.54)	(0.56)	(0.79)	(0.76)	(0.76)	(0.86)
	No (*N*= 249)	3.13	2.95	2.93	3.10	2.81	3.81	3.62	3.54	3.84
		(0.55)	(0.51)	(0.51)	(0.55)	(0.49)	(0.77)	(0.75)	(0.81)	(0.74)

^a^Scale range 0–4, higher score indicates greater ability or more support; ^b^Scale range 0–5, higher score indicates greater ability or more support. Variables in bold are significant at the 0.05 level by the Mann–Whitney U test for non-parametric data and one-way ANOVA. ^c^Effect size (ES) calculated using Cohen’s d for standardised difference in means. Interpretation of ES: “small” ES >0.20-0.50 SD identified by ^d^, “medium” ES 0.50-0.80 SD identified by ^e^, and “large” ES >0.80 SD identified by ^f^

Of the three conventional indicators of socioeconomic status (education, employment status and income), education showed the largest group differences for some variables. The only consistent finding across all three indicators was that participants with lower socioeconomic status reported more difficulty (lower scores) in *Ability to find good health information* (Education ES 0.38, Occupational Status 0.37 and Income 0.32). We found no relationship between any of the HLQ scales and private health insurance status.

There did not appear to be an association between greater use of hospital services and lower scores across any of the health literacy scales (Table 5). Having three or more hospital admissions at the index hospital in the past 12 months was associated with higher scores for *Feeling understood and supported by healthcare providers* and *Social support for health*, while a greater number of self-reported ED presentations in the last 12 months to any hospital was associated with higher scores for *Active engagement with healthcare* and *Navigating the healthcare system*. No differences in health literacy were seen based upon length of hospital stay (<7 days vs ≥7 days) or ED presentations (<2 versus ≥2 ED presentations). As no strong differences were found in these comparisons, multiple regression with adjustment for sociodemographic variables, was not undertaken.

**Table 5:** Association between health literacy and hospital service use

*n*		Healthcare provider support^a^	Having sufficient information^a^	Actively managing health^a^	Social support for health^a^	Active appraisal of health information^a^	Active engagement with healthcare^b^	Navigating the healthcare system^b^	Ability to find good health information^b^	Understanding health information^b^
		Mean (ES)^c^	Mean (ES)^c^	Mean (ES)^c^	Mean (ES)^c^	Mean (ES)^c^	Mean (ES)^c^	Mean (ES)^c^	Mean (ES)^c^	Mean (ES)^c^
Self-reported non-urgent ED attendance last 12 months (any hospital)	Yes (*N* = 340)	3.14^d^	2.97^d^	2.94^d^	3.12^d^	2.82	**3.84** ^**d**^	**3.65** ^**d**^	3.56	3.85^d^
		(0.57)	(0.52)	(0.52)	(0.55)	(0.52)	**(0.78)**	**(0.74)**	(0.80)	(0.78)
	No (*N* = 32)	2.97^d^	2.84^d^	2.80^d^	2.98^d^	2.80	**3.56** ^**d**^	**3.32** ^**d**^	3.45	3.70^d^
		(0.10)	(0.48)	(0.55)	(0.47)	(0.56)	**(0.81)**	**(0.79)**	(0.80)	(0.78)
Length of stay <7 days	Yes (*N* = 332)	3.13	2.98^d^	2.94	3.11	2.82	3.82	3.63	3.57	3.86
		(0.56)	(0.51)	(0.52)	(0.54)	(0.51)	(0.79)	(0.77)	(0.80)	(0.78)
	No (*N* = 48)	3.09	2.86^d^	2.88	3.07	2.80	3.79	3.63	3.48	3.72
		(0.62)	(0.53)	(0.53)	(0.61)	(0.58)	(0.72)	(0.64)	(0.82)	(0.75)
≥3 admissions in last 12 months in this hospital	Yes (*N* = 28)	**3.38** ^**d**^	3.11^d^	3.09^d^	**3.30** ^**d**^	2.85	3.88	3.64	3.46	3.88
		**(0.48)**	(0.55)	(0.42)	**(0.58)**	(0.56)	(0.72)	(0.82)	(0.78)	(0.70)
	No (*N* = 350)	**3.11** ^**d**^	2.95^d^	2.92^d^	**3.09** ^**d**^	2.82	3.81	3.63	3.56	3.84
		**(0.57)**	(0.51)	(0.53)	**(0.54)**	(0.52)	(0.78)	(0.75)	(0.80)	(0.79)
≥2 non-urgent ED presentations in last 12 months in this hospital	Yes (*N* = 45)	3.10	3.02	3.02^d^	3.19	2.88	3.72	3.59	3.48	3.81
		(0.64)	(0.61)	(0.59)	(0.65)	(0.60)	(0.90)	(0.89)	(0.98)	(0.89)
	No (*N* = 334)	3.13	2.96	2.92^d^	3.10	2.81	3.83	3.63	3.56	3.85
		(0.56)	(0.50)	(0.51)	(0.53)	(0.51)	(0.76)	(0.73)	(0.77)	(0.77)

^a^Scale range 0–4, higher score indicates greater ability or more support; ^b^Scale range 0–5, higher score indicates greater ability or more support. Variables in bold are significant at the 0.05 level by the Mann–Whitney U test for non-parametric data and one-way ANOVA. ^c^Effect size (ES) calculated using Cohen’s d for standardised difference in means. ^d^‘Small’ effect size (ES) > 0.20-0.50

## Discussion

We identified a range of potentially modifiable health literacy needs in hospitalised patients. Consistent with our hypotheses, we found associations between increased age, coming from CALD background, having lower education and lower socioeconomic status, little use of the Internet and lower scores on some health literacy scales. Age, employment status, receiving means-tested government benefits and being from a CALD background were associated with increased use of some hospital services. However the data did not support our hypothesis, or previous studies [15–17, 26], that low health literacy would be associated with greater use of hospital services. On the contrary, we found that people with higher scores for two aspects of health literacy, *Feeling understood and supported by healthcare providers* and *Social support for health* were likely to be more frequent users of hospital services.

There is an extensive body of research that has demonstrated associations between being from a CALD background, lower health literacy, and health outcomes, including increased hospital utilisation [21, 27–31]. Being from a CALD background not only presents challenges for people in finding and understanding relevant and culturally appropriate health information and support in their language, but also presents challenges around differing health care delivery contexts. Our finding of an association between being born in a country where English was not the primary language and lower health literacy may correspond to differences in health care contexts (particularly pertinent for navigating the healthcare system), and differences in childhood opportunity, with these individuals likely to be migrants or descendants of migrants whose educational attainment and income opportunity may have been limited [32]. Our finding of an association between increased age, lower educational attainment and low socioeconomic status and lower health literacy is also in keeping with findings from previous studies [30, 31, 33–35]. However, unlike the prevailing literature [26–28], we did not find being from a CALD background was associated with increased use of hospital services – in fact we found that people from a CALD background were more likely to report less attendances at any ED over the last 12 month months.

Our finding of an association between older age, lower educational attainment and low socioeconomic status and lower health literacy is also in keeping with findings from previous studies [30, 31, 33, 35]. Our findings extend previous work by providing information on the relative strengths of these associations using effect sizes.

In contrast to previous studies [15–17], we did not observe an association between lower scores across any of the HLQ scales and higher use of hospital services. Several factors may explain these differences. We used a multi-dimensional self-report measure of health literacy whereas previous studies used functional health literacy tests that directly assess health-related reading, comprehension and numeracy. The correlation between these different measures has been found to be low. It is possible that the participants in our study, who all have free access to hospital services as part of the Australian healthcare system, are in an environment of high disease burden and overall low health literacy. It may also be that health literacy is irrelevant to individuals who require timely acute hospital-based care. As health literacy is a multidimensional concept, it may also be that looking for associations between individual scales of health literacy and use of hospital services is less relevant than considering an individual as a whole as strengths in some scales may offset limitations in other scales. Further investigation using different analytic techniques such as grouping individuals on the basis of specific health literacy levels may shed new light on these relationships.

Our study has several limitations. Our low response rate may limit the generalisability of our results to the broader hospitalised population, however we expect the data to have reasonable internal validity. Importantly, age, gender, reason for admission and use of hospital services were similar between respondents and non-respondents, suggesting minimal response bias. Low response rates for mailed surveys in low socioeconomic populations show that those who return surveys are more likely to have higher socioeconomic indicators and health literacy [35–38]. This implies that our study may have underrepresented individuals with low and very low health literacy. While our data are internally valid, underrepresentation of people with low and very low health literacy may also have limited our ability to detect a relationship, if it exists, between health literacy and hospital services use. While availability of the HLQ in several languages likely improved the response rate from people with CALD backgrounds, with the exception of Chinese respondents, the response rate was still lower for these groups. Further, while care was taken in the translation of the HLQ to other languages, there is still some possibility that the intended meaning of items was conceptualised differently in groups from different cultures resulting in potential response bias [39]. Future research should consider alternative methods (such as face-to-face) for collecting data from CALD populations.

Our study also has some strengths. Use of the HLQ to measure health literacy provides insight across a broad range of domains of health literacy in addition to an individual’s health-related reading, comprehension and numeracy skills, which has been the focus of many past studies utilizing functional health literacy instruments [35]. We also used Cohen’s effect sizes to improve the understanding of the relative strength of associations. This information assists with making decisions about where health literacy interventions should be focused to have the largest impact.

This study has provided new insights into the complexity of health literacy, including not only an individual’s skills in terms of finding, understanding and using health-related information but also their ability to navigate the health system and engage with health professionals, and their social supports. The HLQ embraces the full concept of health literacy by encompassing measurement of both a set of different individual skills and the lived experience of a person interacting with the services, systems and environment. These need to be considered together. For example, we found that increased age was associated with greater difficulty in finding health information, but older individuals were also more likely to report greater healthcare provider and social support than those under age 65 years. This suggests that social and health professional supports can offset other health literacy difficulties, while lack of these supports might indicate a need to provide additional assistance and resources.

## Conclusion

Using a panel of nine fine-grained indicators of health literacy (the HLQ), this study did not find an association between lower health literacy and greater use of hospital health services. However we did find that increased age (≥65 years), having a CALD background and not speaking English at home were all associated with having the most health literacy challenges, particularly around engaging with and feeling supported by health care providers, navigating the health system and finding and using health information. Strategies are needed that address these health literacy needs and should be evaluated to determine if they improve the quality of care and improve patient-relevant outcomes including reducing avoidable admissions. This type of approach may also reduce health inequalities.

## Abbreviations

CALD: Culturally and linguistically diverse
ED: Emergency department
HLQ: Health Literacy Questionnaire
